# Interleukin-34 inhibits hepatitis B virus replication *in vitro* and *in vivo*

**DOI:** 10.1371/journal.pone.0179605

**Published:** 2017-06-14

**Authors:** Sheng-Tao Cheng, Hua Tang, Ji-Hua Ren, Xiang Chen, Ai-Long Huang, Juan Chen

**Affiliations:** 1 Key Laboratory of Molecular Biology for Infectious Diseases (Ministry of Education), Institute for Viral Hepatitis, Department of Infectious Diseases, The Second Affiliated Hospital, Chongqing Medical University, Chongqing, China; 2 Department of Clinical Laboratory, Zhuzhou Central Hospital, Zhuzhou, China; 3 Collaborative Innovation Center for Diagnosis and Treatment of Infectious Diseases, Zhejiang University, Zhejiang, China; Academia Sinica, TAIWAN

## Abstract

**Background:**

The hepatitis B virus (HBV) infection could activate the immune system and induce extensive inflammatory response. As the most important inflammatory factor, interleukins are critical for anti-viral immunity. Here we investigated whether interleukin-34 (IL-34) play a role in HBV infection.

**Methodology/Principal findings:**

In this study, we first found that both serum IL-34 and IL-34 mRNA in PBMCs in chronic HBV patients was significantly decreased compared to the healthy controls. Furthermore, both IL-34 protein and mRNA levels were declined hepatoma cells expressing HBV. In addition, the clinical parameters analysis found that serum IL-34 was significantly associated with HBV DNA (*P* = 0.0066), ALT (*P* = 0.0327), AST (*P* = 0.0435), TB (*P* = 0.0406), DB (*P* = 0.0368) and AFP (*P* = 0.0225). Correlation analysis also found that serum IL-34 negatively correlated with HBV DNA copies, ALT and AST. In vitro studies found that IL-34 treatment in HepAD38 and HepG2.2.15 cells markedly inhibited HBV DNA, total RNA, 3.5kb mRNA and HBc protein. In vivo studies further demonstrated IL-34 treatment in HBV transgenic mice exhibited greater inhibition on HBV DNA, total RNA, 3.5kb mRNA and HBc protein, suggesting the effect to IL-34 on HBV is likely due to host innate or adaptive immune response.

**Conclusions/Significance:**

Our study identified a novel interleukin, IL-34, which has anti-viral activity in HBV replication in hepatocytes in vitro and in vivo. These data suggest a rationale for the use of IL-34 in the HBV treatment.

## Introduction

Hepatitis B virus (HBV) infection remains a major health problem worldwide [1]. The HBV genome is a relaxed circular partially double-stranded DNA (rcDNA) of approximately 3200 bp [2], which contains four open reading frames: preS/S, pol, PreCore/core and X [3]. Mediated by Na+-taurocholate cotransporting polypeptide (NTCP) and other receptors [4], HBV nucleocapside is released into the cytoplasm and then rcDNA is delivered into the nucleus. In nucleus, rcDNA reverted into covalently closed circular DNA (cccDNA), which contains the complete genetic script of HBV to serve as the transcriptional template [5]. cccDNA persistence within hepatocytes is the main reason for the failure of viral clearance and relapse of viral activity after antiviral treatment in clinical [6]. There are two major therapy strategies which are applied for HBV treatment: direct acting antiviral drugs and immune modulatory agents [7]. However, none of current therapies could completely cure HBV infection and more effective therapies are needed for HBV treatment.

As a newly discovered member of interleukin family which shows no sequence similarity with macrophage colony stimulating factor (CSF-1) [8], IL-34 has received much attention. In physiological condition, IL-34 promotes differentiation, proliferation and survival of mononuclear cells via strongly binding to CSF-1R [9] [10]. Dysregulation of the IL-34 is reported to be involved in many diseases, such as inflammatory bowel disease [11], rheumatoid arthritis [12], heart failure [13] and kidney diseases [14]. In terms of virus infection, IL-34 could response to influenza A virus (IAV) infection through the inflammatory cascade [15]. Meanwhile, IL-34 enhanced human immunodeficiency virus-1 (HIV-1) production by microglia [16], although the interventional studies in vivo are lacking. It also reported that IL-34 was upregulated in hepatitis C virus infection and inhibited the production of IFN-γ [17]. However, the understanding of the role of IL-34 during HBV infection remains elusive.

To assess the potential role of IL-34 in HBV infection, we investigated the expression and clinical significance of IL-34 in patients with chronic HBV infection. And further studies indicated that IL-34 may inhibit HBV replication both in *vitro* and in *vivo*. Those results improve our understanding of the underlying mechanisms by which IL-34 regulates HBV replication.

## Materials and methods

### Patients selection

With informed consent from patients, blood specimens were obtained from 79 patients with chronic hepatitis B virus infection including 42 hepatitis B virus e antigen (HBeAg) positive and 37 HBeAg negative ones. All patients were hepatitis B virus s antigen (HBsAg) positive. Exclusion criteria: the patients with hepatitis C virus, hepatitis D virus, human immunodeficiency virus type 1 [HIV-1], and HIV-2, and other causes of chronic liver damages. These patients were not suffering from any autoimmune disease. Clinicopathologic and outcomes data were collected retrospectively. 105 sex- and age-matched healthy Chinese volunteers were recruited as normal control subjects. The Clinical Research Ethics Committee of the Chongqing Medical University approved the above protocol.

### Antibodies, plasmids, and drug

HBV core antibody (B0586) was obtained from Dako (Dako, Berchem, Denmark). Rabbit anti-HBcAg polyclonal antibody was obtained from Dako (Glostrup, Denmark). Mouse anti-GAPDH monoclonal antibody was purchased from Zhongshan Golden Brige Biotechnology (ZSGB-Bio, China). HBV replication plasmid pCH9/3091 which contains 1.1 copies of the HBV genome, was kindly provided by Prof. Lin Lan (The Third Military Medical University, Chongqing, China). Recombinant human interleukin-34 (RhIL-34) was purchased from R&D Systems (Catalog no. 5265-IL-010, Minneapolis, MN).

### Cell culture and drug treatment

HepAD38 cell was purchased from the Shanghai Second Military Medical University and cultured in modified Eagle medium (MEM) (Catalog no. 11960046, Corning, New York, USA) with 10% fetal bovine serum (FBS) (Catalog no. 10270, Corning, New York, USA), 1% sodium pyruvate (Catalog no. 1752424, Gbico, USA) and 400 ug of G418 (Catalog no. 345810, Merck, Germany) per ml. HepG2.2.15 cell was purchased from the Shanghai Second Military Medical University and cultured in Dulbecco’s modified Eagle medium (DMEM) (Catalog no. 10-010-CVR, Corning, New York, USA) with 10% fetal bovine serum (FBS). Huh-7 was acquired from the Health Science Research Resource Bank (Osaka, Japan) and were cultured in Dulbecco’s modified Eagle medium (DMEM) with 10% fetal bovine serum (FBS). All the cells were maintained in a humidified incubator at 37°C with 5% CO_2_. The cells were seeded into 6-well plates and treated with different concentrations of IL-34 (0 ng/ml, 20 ng/ml, 40 ng/ml). After 3 or 5 days of treatment, total RNA, protein, or HBV replicative intermediates were collected.

### Animals

HBV transgenic mice (HBV-Tg C57BL/6) were kindly provided by Prof. Ningshao Xia (Xia Men University, China). A 1.2-overlength copy of the HBV genome (serotype awy) was encoded in these animals. Mouse were bred and maintained under pathogen-free conditions at the Laboratory Animal Center of the Chongqing Medical University. For experiment they were matched for age (6–8 weeks), sex (male), serum HBV DNA and HBV surface antigen (HBsAg) levels. The mice were randomly assigned to two groups of 6–7 individuals per group. RhIL-34 was diluted in 1 ml PBS contained 0.1% BSA and injected through the tail vein at a dose of 1ug/25g. The serum samples were collected via tail vein at the indicated time points after injection. The mice were sacrificed by cervical dislocation to collect liver samples. The animal studies were carried out in accordance with the Chinese Council on Animal Care and approved by Chongqing Medical University. Laboratory Animal Center of the Chongqing Medical University approved the animal studies.

### HBV DNA preparation

HBV replicative intermediates were obtained as described previously [18]. HBV genome DNA in mouse serum and liver were extracted by using TIANamp Virus DNA/RNA Kit (Catalog no. DP315, Tiangen, China) and Wizard Genomic DNA Purification Kit (Catalog no. A1120, Promega, USA), respectively.

### Quantitative real-time PCR (qPCR)

The absolute quantification of the HBV replicative intermediates and mouse serum and liver HBV DNA were detected using Fast Start Universal SYBR Green Master (Catalog no. 06924204001, Roche, Mannheim, Germany). Serial dilutions of HBV DNA plasmids were used to standardize the results. IScript^™^ cDNA Synthesis Kit was purchased from Bio-Rad (Catalog no. KR106-02, Bio-Rad, California, USA). Relative quantification of HBV total RNA and 3.5kb mRNA were conducted using Fast Start Universal SYBR Green Master and β-actin mRNA was used as an internal control. The fold change of target genes were calculated by using the 2-ΔΔCT method. The sequences of the experimental primers are as follows: HBV replicative intermediates: forward, 5’-CCTAGTAGTCAG TTATGTCAAC-3’, reverse, 5’-TCTATAAGCTGGAGGAGTGCGA-3’. Mouse serum and liver HBV DNA: forward, 5’- CCTCTTCATCCTGCTGCT-3’; reverse, 5’- AACTGAAAGCCAAACAGTG-3’. HBV total RNA: forward, 5’-ACCGACCTTGAGGCATACTT-3’, reverse, 5’- GCCTACAGCCTCCTAGTACA-3’. HBV 3.5kb mRNA: forward, 5’- GCCTTAGAGTCTCCTGAGCA-3’ reverse, 5’- GAGGGAGTTCTTCTTCTAGG-3’. β-actin: forward, 5’-CTCTTCCAGCCTTCCTTCCT-3’, reverse, 5’-AGCACTGTGTTGGCGTACAG-3’.

### Enzyme-linked immunosorbent assay (ELISA)

Serum levels of IL-34 in chronic HBV infection patients were determined by IL-34 ELISA kits (Catalog no. JYM2045Hu, ColorfulGene Biological Technology, Wuhan, China) according to the manufacturer’s instructions.

### Southern blot

The HBV DNA replicative intermediates were separated by 0.9% agarose gels and denatured in alkali solution at room temperature for 30 min. And then the DNA was transferred on nylon membrane by capillary siphon method and fixed by UV cross-linking. After pre-hybridization at 42°C for 1 h, the membrane was hybridized with digoxigenin-labeled DNA probe overnight at 42°C. The next day, the membrane was washed in 30 ml of 2 x SSC/0.1% SDS, 1 x SSC/0.1% SDS, 0.1 x SSC/0.1% SDS for 15 min, respectively. The membrane was blocked at 37°C for 30 min in blocking solution and incubated with anti-digoxin secondary antibody at 37°C for 30 min. The DIG-High Prime DNA Labeling and Detection Starter Kit (Catalog no. 11585614910, Roche, Mannheim, Germany) was used in this experiment. The signal was collected by X-ray film.

### Northern blot

HBV RNAs were analyzed according to DIG Northern Starter Kit (Catalog no. 12039672910, Roche, Switzerland) manufacturer’s protocol. The extracted RNA was separated by 1.4% formaldehyde-agarose gel and was stained with ethidium bromide to evaluate the quality of the target RNA under UV light. The RNA was transferred on nylon membrane by capillary siphon method. After pre-hybridization, the membrane was hybridized with digoxigenin-labeled RNA probe overnight. Then the membrane was incubated in blocking solution and antibody solution at 37°C for 30 min, respectively. Finally, the signal was collected by X-ray film.

### Western blot

The cells and tissues were collected and lysed with appropriate volume of RIPA lysis buffer containing protease inhibitor. The protein was quantified by BCA (Catalog no. 23223, Roche, Mannheim, Germany) and the lysates containing 30 ug of total protein was denatured at 95°C for 10 min. After separated by SDS-PAGE, the protein was transferred to nitrocellulose membrane (Catalog no. RPN303D, GE Healthcare, Buckinghamshire, UK) by electric wet transfer method. The membrane containing the protein of interest was blocked in 5% nonfat milk and incubated with primary antibody (Anti-HBV core protein 1:800; Anti-GAPDH 1:10000) overnight at 4°C. The next day the membrane was washed in TBS-T for 5 min, and repeated three times. The secondary antibody was incubated at room temperature for 2 h on a shaker (antibody 1: 3000 dilution). The bands were visualized with ECL Western blot reagents (Catalog no. WBKLS0500, Millipore, Massachusetts, USA). GAPDH was used as a loading control.

### Serum ALT and AST detection

Serum alanine transaminase (ALT) and aspartate transaminase (AST) in mouse treated with IL-34 or control were measured using commercial kits purchased from Nanjing Jiancheng Bioengineering Institute (ALT: Catalog no. C009-2, AST: Catalog no. C010-2, Jiancheng, Jiangsu, China) according to the manufacturer’s protocols.

### Statistical analysis

Results are expressed as mean±SD. IL-34 expression in chronic HBV patients and healthy controls were compared by the Student’s t-test. Correlations between IL-34 and clinicopathologic parameters were analyzed by nonparametric χ^2^ test and Spearman’s rank test. A difference was considered significant when *P*<0.05. All statistical analysis was performed by the SPSS 19.0 software.

## Results

### Characteristics of chronic HBV patients and healthy control subjects

This study recruited 79 chronic HBV patients and 105 age- and sex-matched healthy controls. The clinical and virologic characteristics of all the subjects were described in Table 1. The serum concentrations of total protein, albumin, alkanine aminotransferase (ALT), aspartate aminotransferase (AST), total bilirubin (TB), direct bilirubin (DB) and alpha-fetoprotein (AFP) were significantly elevated in chronic HBV patient group compared to the healthy control group, indicating that the liver damage caused by HBV infection.

**Table 1:** Clinical and virological characteristics of the subjects enrolled in the study.

Baseline characteristics	Healthy controls (n = 105)	Chronic hepatitis B (n = 79)	*P* value
Age, years [median (IQ range)]	40 (23–61)	41 (18–69)	ns
Male/female	55/50	41/38	ns
HBsAg positive	0/105	79/79	
HBeAg positive	0/105	42/79	
HBcAb positive	0/105	5/79	
Cirrhosis positive	0/105	28/79	
Total protein g/L [median (IQ range)]	73.4 (61.3–82.6)	70.1 (49.2–91.5)	<0.0001
Albumin g/L [median (IQ range)]	50.2 (45.1–55)	38.5 (25.4–48.5)	<0.0001
ALT, U/L [median (IQ range)]	17.1 (9.0–49.0)	493.0 (19.0–1792.0)	<0.0001
AST, U/L [median (IQ range)]	20.7 (15.0–38.0)	197.0 (19.0–1539.0)	<0.0001
TB, μmol/L [median (IQ range)]	11.44 (4.80–19.29)	30.50 (5.50–713.00)	<0.0001
DB, μmol/L [median (IQ range)]	2.84 (1.20–10.37)	26.20 (1.50–437.00)	<0.0001
AFP, ug/L [median (IQ range)]	0.31 (0.01–12.49)	24.50 (2.01–396.10)	<0.0001
HBV DNA log10 (IU/ml)	0	5.74 (3.30–9.24)	
Autoimmune diseases	None	None	

IQ: interquartile, TP: total protein, ALT: alkanine aminotransferase, AST: aspartate aminotransferase, TB: total bilirubin, DB: direct bilirubin, AFP: Alpha-fetoprotein. n: number of individuals. *P* when compared with healthy controls.

### IL-34 expression in HBV infection patients and HBV-expression cells

**Fig 1:**
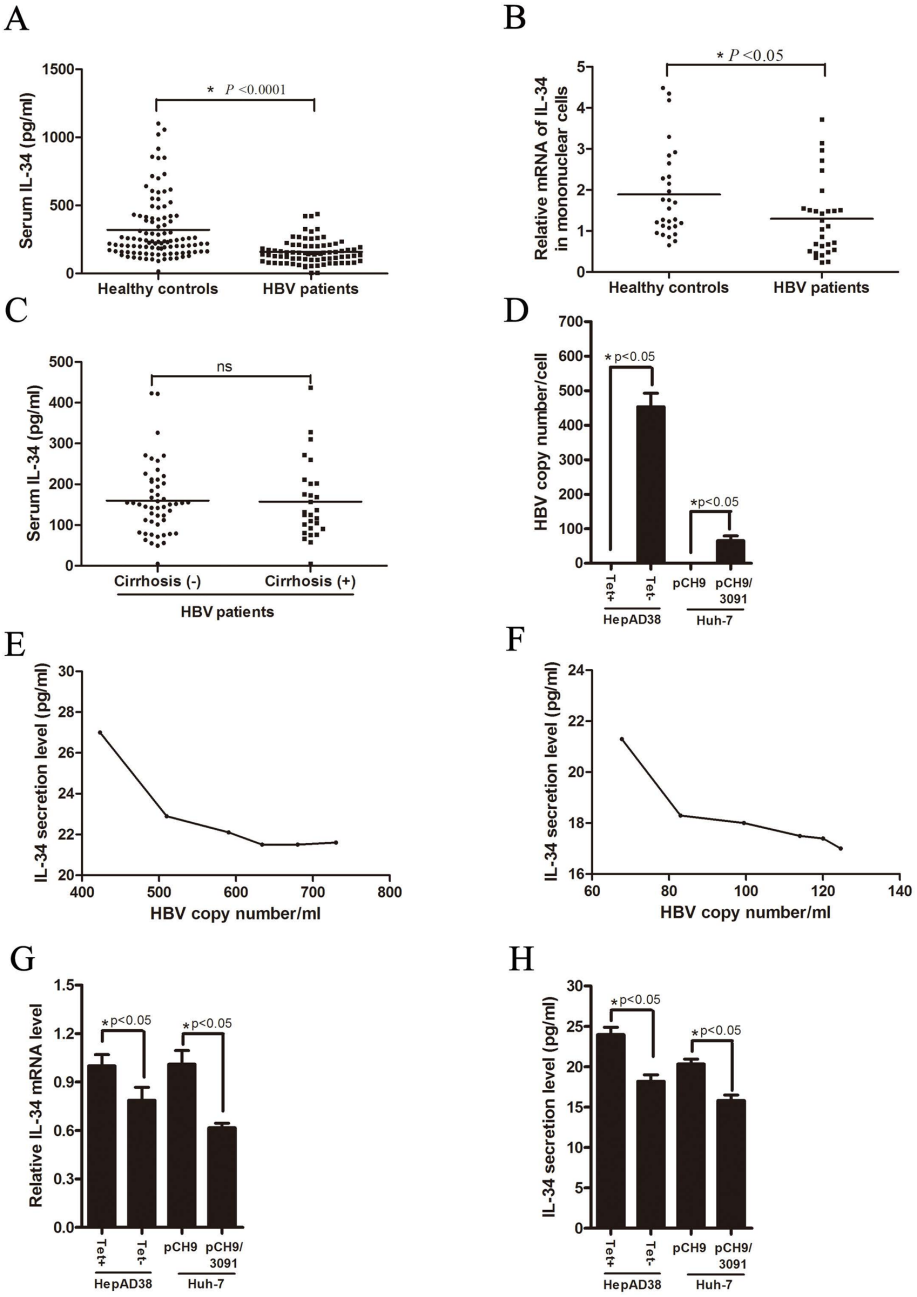
Secretion concentrations of IL-34 in HBV patients and HBV-expressing cells. (A) The serum levels of IL-34 in chronic HBV patients and healthy controls were determined by ELISA kits, * *p*<0.0001. (B) The peripheral blood mononuclear cells (PBMCs) in chronic HBV patients and healthy controls were isolated from full blood and total RNA were extracted. Then the mRNA level of IL-34 were determined by quantitative real-time PCR (qPCR), * *p*<0.05. The mRNA level of β-actin was used as an internal control. (C) The levels of serum IL-34 in chronic HBV patients with or without cirrhosis were determined by ELISA kits, ns: no significance. (D) QPCR were used to determine the HBV copy numbers in HepAD38 cells with tetracycline (no HBV replication) and without tetracycline (HBV replication) and Huh-7 cells transiently transfected with plasmid pCH9/3091 or control vector pCH9. (E-F) The supernatants of HepAD38 without tetracycline (HBV replication) and Huh-7 cells transiently transfected with plasmid pCH9/3091 post seeded or transfected 2 days, 3 days, 4 days, 5 days, 6 days and 7 days were collected and then the HBV DNA or IL-34 secretion level were detected by qPCR or ELISA kits. (G) The mRNA levels of IL-34 in HepAD38 cells (With or without tetracycline) and Huh-7 cells transiently transfected with plasmid pCH9/3091 or pCH9 were determined by quantitative real-time PCR (qPCR), * *p*<0.05. The mRNA level of β-actin was used as an internal control. (H) Supernatants of HepAD38 cells (with or without tetracycline) and Huh-7 cells transiently transfected with plasmid pCH9/3091 or pCH9 were collected and secretion concentrations of IL-34 were determined by ELISA kits, * *p*<0.05.

To explore whether IL-34 plays a role in the pathogenesis of chronic HBV infection, we first determined the serum concentration of IL-34 in chronic HBV patients and healthy controls by using ELISA (Fig 1A). The level of serum IL-34 in chronic HBV patients (median: 145.00 pg/ml) were significantly decreased compared to those of healthy controls (median: 230.43 pg/ml), suggesting that IL-34 may play a potential role in HBV pathogenesis. To further confirm this hypothesis, the peripheral blood mononuclear cells (PBMCs) was isolated from blood samples of 29 pairs HBV patients and healthy controls and the levels of mRNA of IL-34 were determined by quantitative real-time PCR (qPCR) (Fig 1B). Consistently, IL-34 mRNA level in PBMCs decreased in chronic HBV patients relative to healthy controls. Those data indicated that IL-34 indeed plays a potential role in HBV infection *in vivo*. In order to clarify the impact of disease progression on IL-34 levels, we divided HBV patients into two groups (With or Without cirrhosis) based on pathological findings and further compared the serum IL-34 levels between those two groups (Fig 1C). According to our results, cirrhosis status did not have significant effect on IL-34 expression.

To further investigate the association between HBV and IL-34, we analyzed the IL-34 mRNA level in HepAD38 and the corresponding cell culture mediums were collected to analyze the secretion levels of IL-34 (Fig 1D–1H). HepAD38 is HBV stably expressing cells where HBV genome integrates host genome and the expression of HBV can be regulated by tetracycline. HBV expression is inhibited when tetracycline is present in HepAD38 cell culture medium. To certificate the HBV expression in HepAD38 cells were inhibited by tetracycline, HBV DNA in HepAD38 cells with or without tetracycline were detected by qPCR (Fig 1D). The supernatants of HepAD38 without tetracycline (HBV replication) in 2 days, 3 days, 4 days, 5 days, 6 days and 7 days were collected and then the HBV DNA or IL-34 secretion level were detected. With the increase of HBV copy number, the secretion level of IL-34 decreased (Fig 1E). Both the mRNA and secretion level of IL-34 were down regulated in HepAD38 cells without tetracycline compared to cells with tetracycline treatment (Fig 1G–1H).

The expression of IL-34 also examined in Huh-7 cells transiently transfected HBV expressing plasmid pCH9/3091. QPCR were used to determine the HBV replication intermediate in Huh-7 cells transiently transfected pCH9/3091 (Fig 1D). The supernatants of Huh-7 cells transiently transfected HBV expressing plasmid pCH9/3091 in 2 days, 3 days, 4 days, 5 days, 6 days and 7 days were also collected and then the HBV DNA or IL-34 secretion level were detected. IL-34 protein level decreased with increasing HBV copy number (Fig 1F). Similarly, both the mRNA and secretion level of IL-34 in Huh-7 cells transfected with pCH9/3091 were decreased compared the control cells transfected with vector (Fig 1G–1H).

### Correlation of clinical parameters with IL-34 levels in chronic HBV patients

Based on the finding that the serum levels of IL-34 were significantly decreased in chronic HBV patients, the clinicopathologic and outcomes data of those patients were collected retrospectively. According to the serum level of IL-34, chronic HBV patients were further classified into low and high expression of IL-34 (low expression: IL-34<150 pg/ml, high expression: Il-34>150 pg/ml). Among the 79 cases, 40 showed low expression, and 39 showed high expression (Table 2). IL-34 expression levels showed association with many clinical parameters, including HBV DNA (*P* = 0.0066), ALT (*P* = 0.0327), AST (*P* = 0.0435), TB (*P* = 0.0406), DB (*P* = 0.0368) and AFP (*P* = 0.0225).

**Table 2:** Correlative analysis of serum IL-34 level with clincopathologic features in chronic HBV patients.

Clincopathologic features	IL-34 Expression		*P* value
	Low (n = 40)	High (n = 39)	
HBeAg			
Negative	23	19	0.5021
Positive	17	20	
ALT, U/L			
< 450	9	19	0.0327
> 450	31	20	
AST, U/L			
< 200	25	15	0.0435
> 200	15	24	
TB, μmol/L			
< 140	34	25	0.0406
> 140	6	14	
DB, μmol/L			
< 60	30	20	0.0368
> 60	10	19	
AFP, ug/L			
< 40	29	18	0.0225
> 40	11	21	
HBV DNA log10 (IU/ml)			
< 4	6	17	0.0066
> 4	34	22	

**Fig 2:**
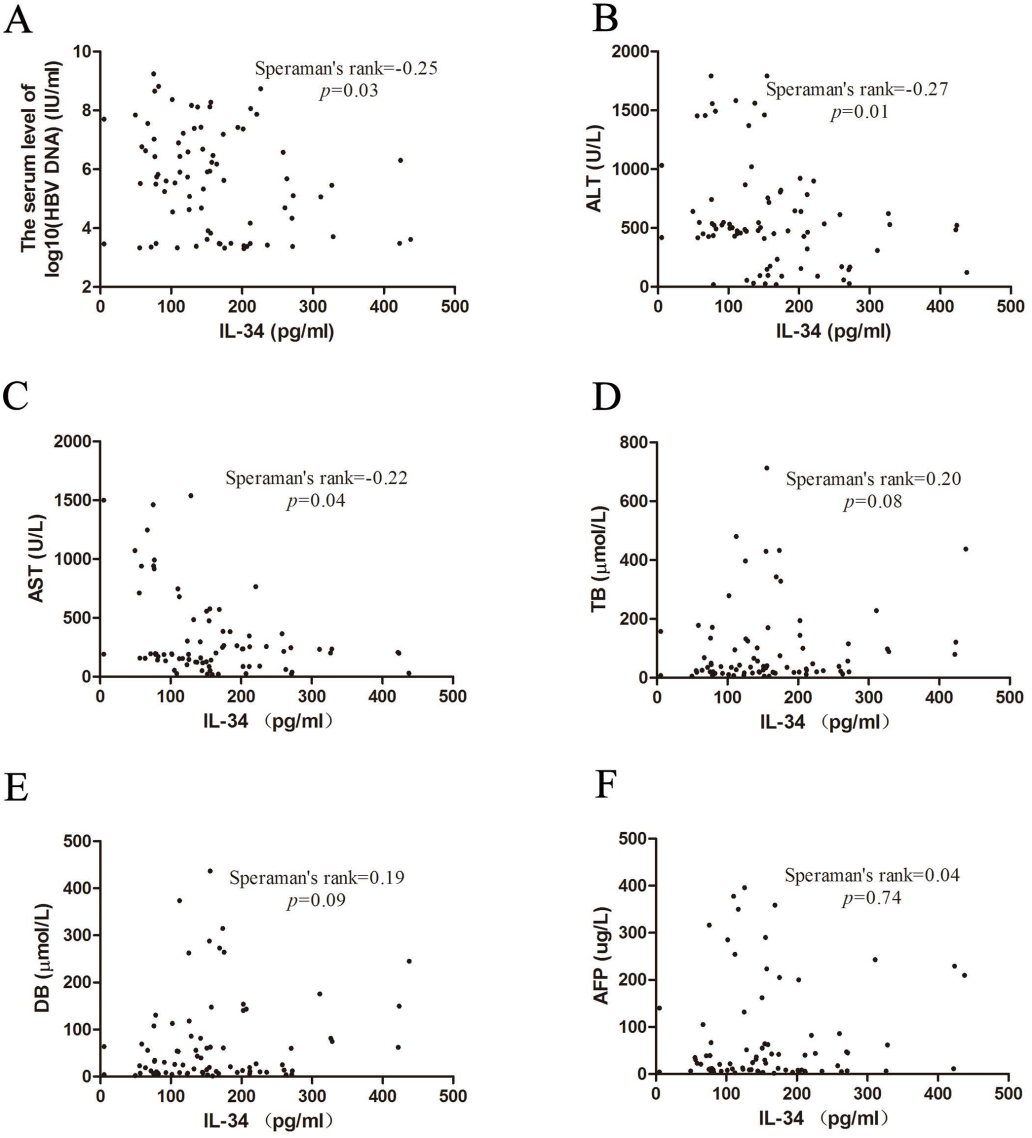
Correlation of clinical parameters with IL-34 levels in chronic HBV patients. The clinicopathologic and outcomes data of those patients were collected retrospectively. The correlations of HBV DNA (A), ALT (B), AST (C), TB (D), DB (E) and AFP (F) with IL-34 levels were further analyzed by Spearman’s rank test.

The correlations of clinical parameters with IL-34 levels were further analyzed. We found that serum IL-34 negatively correlated with the serum level of HBV DNA (log10(HBV DNA)) (Fig 2A, Speraman’s rank = -0.25, *P* = 0.03) in chronic HBV patients. Moreover, ALT and AST, which were liver injury markers, also been analyzed. Serum IL-34 negatively correlated with ALT and AST (Fig 2B and 2C, Speraman’s rank = -0.27, *P* = 0.01; Speraman’s rank = -0.22, *P* = 0.04, respectively). However, we found that serum IL-34 concentrations did not significantly correlate with TB, DB or AFP in chronic HBV patients (Fig 2D–2F). Taken together, those data suggested that IL-34 down regulation is associated with liver damage.

### IL-34 inhibited HBV replication in vitro

**Fig 3:**
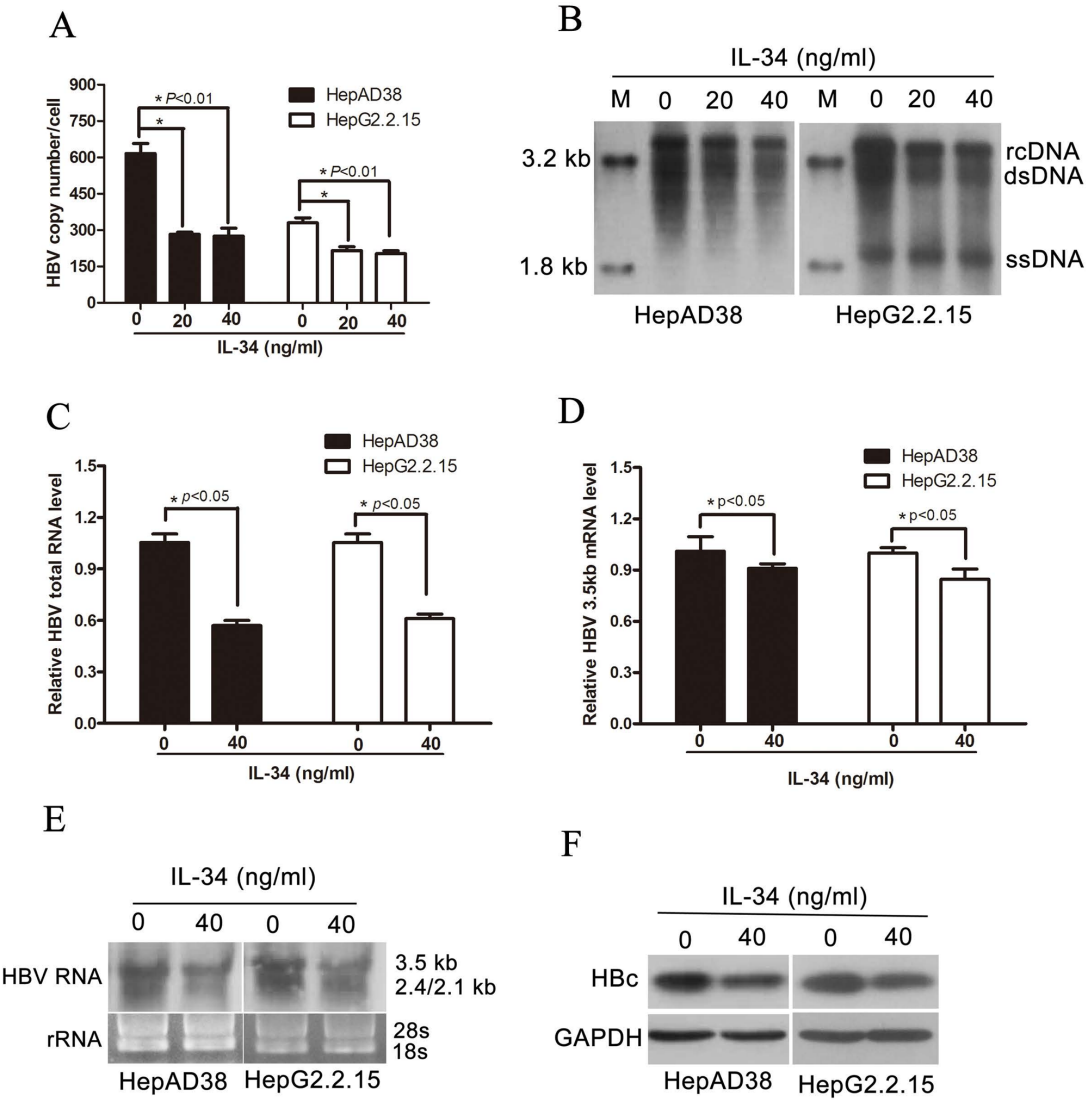
IL-34 inhibited HBV replication in *vitro*. HepAD38 and HepG2.2.15 cells were seeded into 6-well plates and treated with different concentrations of rhIL-34. After 3 or 5 days of treatment, total RNA, protein, or HBV replicative intermediates were extracted. (A-B) rhIL-34 treatment inhibited the production of HBV replicative intermediates. The absolute quantification PCR and Southern blot were performed to determine the copies of HBV replicative intermediates after 5 days treatment, *p<0.01. (C-D) Total RNA was extracted after 3 days treatment. Relative real-time PCR was subjected to detect the HBV total RNA and 3.5kb mRNA levels, * *p*<0.05. The mRNA level of β-actin was used as an internal control. (E) Northern blot was applied to determine the HBV total RNA and 3.5kb mRNA levels. The rRNA level of 28s/18s were used as an internal control. (F) Western blotting analysis of HBc expression after 3 days treatment.

To elucidate the functional role of IL-34 in HBV replication, HepAD38 cells without tetracycline and HepG2.2.15 cells were treated with different concentrations of rhIL-34. RhIL-34 treatment resulted in decreased level of HBV DNA replicative intermediates significantly as evidenced by both real-time PCR and Southern blot (Fig 3A and 3B). HBV 3.5kb mRNA contains all the genetic information of the HBV genome and is a template for HBV replication. Furthermore, total RNA and 3.5kb mRNA were analyzed by qPCR (Fig 3C and 3D). Northern blot was performed to verify the decreased level of HBV total RNAs and 3.5kb mRNA in HepAD38 and HepG2.2.15 cells treated with rhIL-34 (Fig 3E). In addition, HBV core protein was further determined by western blotting analysis. Consistently, rhIL-34 treated markedly inhibited HBV core protein expression (Fig 3F). Together, these data suggested that IL-34 could repress HBV replication.

### IL-34 inhibited HBV replication in transgenic mice

**Fig 4:**
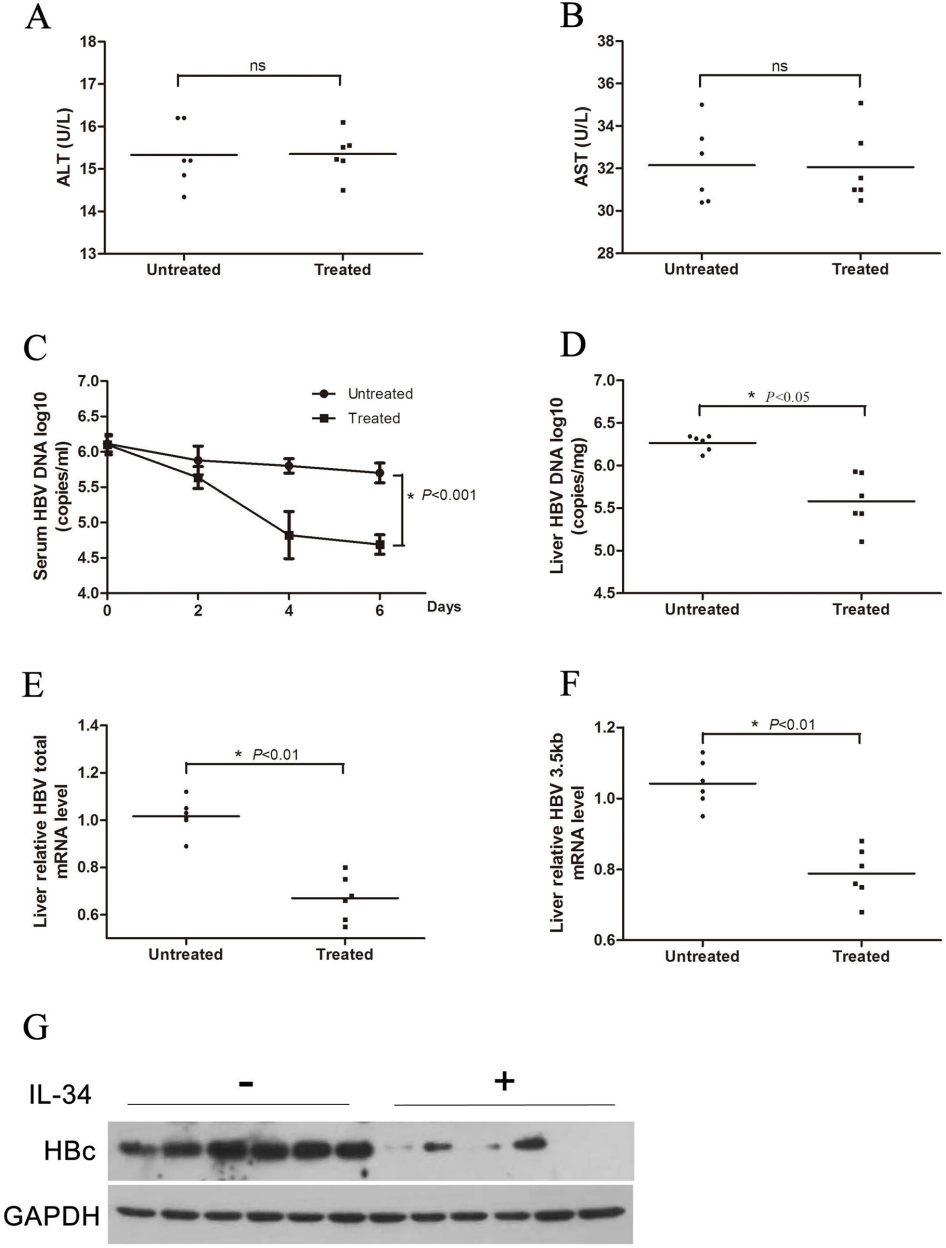
IL-34 inhibited HBV replication in transgenic mice. The mice were randomly assigned to two groups of 6–7 individuals per group. RhIL-34 was diluted in 1 ml PBS contained 0.1% BSA and injected through the tail vein at a dose of 1ug/25g. (A-B) Serum samples at 6 days were collected to detected ALT and AST by Reitman-Frankel methods. (C) Serum samples were collected at the indicated time points (0 day, 2 days, 4 days, 6 days) after injection via tail vein. The serum level of HBV DNA was extracted and analyzed by absolute quantification PCR, * *p*<0.001. (D-G) The mice were sacrificed at 6 days after injection, and the liver was isolated for extraction of HBV DNA, total RNA and total protein. (B) Liver HBV DNA was analyzed by absolute quantification PCR, * *p*<0.05. (E-F) Relative real-time PCR was subjected to detect the HBV total RNA and 3.5kb mRNA levels, * *p*<0.01. The mRNA level of β-actin was used as an internal control. (G) The expression of HBc was analyzed by Western blotting.

To investigate whether IL-34 can inhibit HBV replication in *vivo*, HBV transgenic mice (HBV-Tg C57BL/6) were injected with rhIL-34 solutions with 1ug/25g via tail vein. Mouse serum samples were collected at 0 day, 2 days, 4 days, and 6 days after injection, respectively. All the mice were sacrificed at 6 days, and the liver was isolated for further experiments. To certificate whether there was liver damage after rhIL-34 treatment, we detected the serum level of ALT and AST in HBV transgenic mice receiving IL-34 or PBS contained 0.1% BSA (Fig 4A and 4B). The results showed that ALT and AST levels were not significantly differed between the two groups, indicating that IL-34 treatment did not cause significant liver damage. QPCR analysis showed that serums HBV DNA in IL-34 treated group were decreased in a time dependent manner relative to the control group (Fig 4C). In addition, HBV DNA in mouse liver tissues were also declined in IL-34 treated group compared to control group (Fig 4D). Then HBV total RNA and 3.5kb mRNA were also analyzed. Consistently, IL-34 treatment resulted in decreased HBV total RNA and 3.5kb mRNA (Fig 4E and 4F). HBc in IL-34 treated group was lower than that in control group (Fig 4G) which was detected by Western blot. Collectively, those data as described above suggested that IL-34 had an inhibition effect on HBV replication in transgenic mice.

## Discussion

The main life cycle of HBV including infection, replication, assembly, maturation, and secretion. Based on the extensive research of virus-host interactions, several new agents which aimed to enhance the innate and adaptive immune responses [19] are under development to cure HBV. Interleukins have an irreplaceable role in the immune system and widely involve in varieties of biological processes, such as antibody secretion, interferon-γ (IFN-γ) production, cell proliferation and differentiation [20].

It is well-known that interleukins can affect the biological processes of many viruses. IL-22 do not have significant effect on lethal influenza infection but is beneficial to sublethal infection [21]. IL-32 has a protective role in the immune response to RNA and DNA viruses [22]. More importantly, IL-26 was identified to strongly enhance vesicular stomatitis virus (VSV) infection and replication rates IL-23 could also inhibit human cytomegalovirus (HCMV) and has no effect the herpes simplex virus type 1 (HSV-1) [23]. The above studies strongly indicated that interleukins, even the same one interleukin, plays different role in different virus infection and sometime exert completely different effect. In terms of HBV, IL-4 gene polymorphisms may affect the Korean infants’ response to the HBV vaccine [24]. And the level of IL-23 in monocyte-derived dendritic cells was associated with mortality of acute-on-chronic liver failure (HBV-ACLF) patients [25]. So far, there is no report about the relationship between HBV and IL-34. In this study, we identified that IL-34 was significantly decreased in the serum and PBMCs of chronic HBV patients compared to healthy control subjects.

PBMCs play a central role in immune system responses against microbial infections. A previously study has reported that the dysfunction of peripheral blood mononuclear cell (PBMC) is related to the HBV chronic infection and tolerance [26]. Consistently, our data showed that the mRNA level of IL-34 in PBMCs of chronic HBV patients were obviously decreased compared to healthy subjects. This change would alter the immune response to HBV infection.

To further elucidate the function of IL-34 in HBV infection patients, we studied the role of IL-34 on HBV replication both in *vitro* and in *vivo*. There are complicated interactions between the host immune system and the virus when HBV infected [27]. It is recognized that host immune system responses to HBV invasion to suppress the virus. On the contrary, viruses also alter the immune regulatory effects to escape the recognition and favor their replication [28]. Based on the data in our study, the decreased IL-34 in HBV patients may benefit to HBV replication. In contrast with our study, Yu G, *et al*. reported that IL-34 elevated in influenza A virus infected patients and could be induced by IL-22 in the inflammatory cascade [15]. Considering that IL-34 is recognized as a tissue-restricted ligand of CSF1R [29] and HBV is a hepatotropic virus, the discrepancy between our study and other group may be resulted in from different functions of IL-34 in different tissues. The above findings suggest that IL-34 may play a complicated function on virus infection and could be involved in anti-virus response more than one pathway.

Some studies found that interleukins are involved in pathological processes by regulating microRNAs. IL-21 participates in HIV-1 control in *vivo* via inducing microRNA-29 (miR-29) [30]. And IL-17A would be suppressed by miR-10b to participate the pathological processes of ankylosing spondylitis [31]. Moreover, IL-34 modulates HCC metastasis through microRNA-28 [32]. Therefore, microRNAs may serve as the potential mechanism which IL-34 regulates HBV replication.

As a non-cytolytic virus, HBV can affect host immunization in many ways which is benefit to its own replication. Virus protein HBx can be recruited to the cccDNA and is essential for the transcription of all viral RNAs [33] [34] indicating the important role of HBx to initiate and maintain virus replication. Considering that HBx could downregulate the host antiviral protein APOBEC3G [35] and induce degradation of talin-1 to stimulate HBV replication [36], HBV may regulate IL-34 via HBx. Further studies are needed to elucidate the relationship between HBx and IL-34 and the potential underlining mechanism.

In summary, the current study enhances our understanding of the role of IL-34 in HBV infection. The decreased IL-34 may contribute to the HBV replication which is a potential target of HBV treatment. Further studies are needed to investigate the underlying mechanism of IL-34 to inhibit HBV replication.

### Research and ethical clearance

Written informed consent from each patient enrolled in this study were obtained. The Clinical Research Ethics Committee of the Chongqing Medical University approved the clinical research. The animal studies were carried out in accordance with the Chinese Council on Animal Care and approved by Chongqing Medical University. Laboratory Animal Center of the Chongqing Medical University approved the animal studies.
